# Direct costs of inequalities in health care utilization in Germany 1994 to 2009: a top-down projection

**DOI:** 10.1186/1472-6963-13-271

**Published:** 2013-07-12

**Authors:** Lars Eric Kroll, Thomas Lampert

**Affiliations:** 1 Department of Epidemiology and Health Monitoring, Robert Koch - Institute, Nordufer 20, PO Box 65 02 61 D-13302, Berlin, Germany

**Keywords:** Health care utilization, Health inequalities, Direct costs, Income, Longitudinal data analysis

## Abstract

**Background:**

Social inequalities in health are a characteristic of almost all European Welfare States. It has been estimated, that this is associated with annual costs that amount to approximately 9% of total member state GDP. We investigated the influence of inequalities in German health care utilization on direct medical costs.

**Methods:**

We used longitudinal data from a representative panel study (German Socio-Economic Panel Study) covering 1994 to 2010. The sample consisted of respondents aged 18 years or older. We used additional data from the German Health Interview and Examination Survey for Children and Adolescents, conducted between 2003 and 2006, to report utilization for male and female participants aged from 0 to 17 years. We analyzed inequalities in health care using negative binomial regression models and top-down cost estimates.

**Results:**

Men in the lowest income group (less than 60% of median income) had a 1.3-fold (95% CI: 1.2-1.4) increased number of doctor visits and a 2.2-fold (95% CI: 1.9-2.6) increased number of hospital days per year, when compared with the highest income group; the corresponding differences were 1.1 (95% CI: 1.0-1.1) and 1.3 (95% CI: 1.2-1.5) for women. Depending on the underlying scenario used, direct costs for health care due to health inequalities were increased by approximately 2 billion to 25 billion euros per year. The best case scenario (the whole population is as healthy and uses an equivalent amount of resources as the well-off) would have hypothetically reduced the costs of health care by 16 to 25 billion euros per year.

**Conclusions:**

Our findings indicate that inequalities and inequities in health care utilization exist in Germany, with respect to income position, and are associated with considerable direct costs. Additional research is needed to analyze the indirect costs of health inequalities and to replicate the current findings using different methodologies.

## Background

Social inequalities in health care are a characteristic of almost all European Welfare States [1]. It is well documented that a lower socioeconomic position is strongly associated with a shorter life expectancy, a higher risk for many chronic diseases, and riskier health behaviors [2]. As a result of these differences in health status, the lower socioeconomic strata have used the health care system more often.

For the analyses of trends regarding inequities in health care utilization, Germany is an especially interesting case. First, Germany has a national health insurance scheme with very broad coverage and also widespread coverage of public health insurance despite an optional private insurance for high income earners. Second, co-payments for ambulatory doctor consultations were established during the last two decades, but were banned in 2013; recently payments for hospital days and medication have risen [3,4].

Inequalities in health care utilization are well documented in a number of European countries, including Germany [5,6]. Overall, the results of recent studies suggest that compared with more affluent groups, patients from lower socioeconomic groups tend to visit general practitioners (GP’s) more often, but have less frequent visits to specialists. Inequalities exist in the probability of contact with a doctor and in regard to the number of subsequent visits. Taking into account the indicators for medical treatment need and/or severity of the patients’ health problems, the ‘better off’ tend to utilize GPs and specialists more often. With respect to Germany’s health care system, the results of international comparative studies suggest that Germany has a medium degree of inequality and inequity [5,6].

From an economic perspective, the persisting inequalities in health and the resulting inequalities in health care have created a sizeable footprint in health expenditures; they also pose risks to the productivity of the national economy. In a recent study, Mackenbach and colleagues tried to evaluate the overall costs of health inequalities for Europe [7]. Based on data for inequalities in mortality and self-rated health in the European Union (EU), these authors concluded that about 20% of all health care expenditure is due to the poorer health status of lower socioeconomic groups. When also taking productivity losses and social security expenditures into account, the annual costs amount to approximately 9% of total member state GDP. Following on from this study, further research is needed to qualify whether these results are plausible from a national standpoint, and whether they are persisting over time.

This study aimed to analyze the magnitude and trends of social inequalities in health care utilization in Germany between 1994 and 2009. We used nationally representative data from the German Socio-Economic Panel Study (GSOEP) to analyze the development of increased direct costs caused by health inequalities. We focused on direct costs, because their estimation is, in comparison to the estimation of indirect costs, less dependent on assumptions about lost productivity and/or data on lost life years per social strata (which are not available for Germany). We investigated two consecutive research questions, including (1) the extent of social inequalities within in-patient and out-patient health care, which has been sought in Germany; (2) the costs arising from inequalities in health care.

## Methods

The GSOEP is a longitudinal household panel study that has been conducted annually since 1984 in Western Germany and from 1991 in Eastern Germany [8]. In each participating household, all individuals aged 18 years or older complete a personal questionnaire, typically during spring. The stability of the sample is in excess of 90% for all subsamples; the proportion of household members interviewed in person is about 94% [9]. The study covers a wide range of socioeconomic indicators and a small number of health outcomes. The GSOEP population is regularly updated with new survey samples to reflect changes in the German population. The data have previously been used to analyze socioeconomic inequalities in health and health care [4,10-12]. The GSOEP is approved as being in accordance with the standards of the Federal Republic of Germany for lawful data protection, all participants gave free and informed consent to participate in the survey. The survey ethics are monitored by an independent advisory board at the DIW. We used all the GSOEP waves from 1994 to 2010. The authors signed a contract with the data holders to permit the use and publishing of data for scientific purposes.

Key demographic information about the study sample is shown in Table 1. According to the recommendations of the data holders, we restricted the analysis to men and women aged 18 and above from households who participated twice in the GSOEP. Overall, 36,179 respondents were observed 274,160 times between 1994 and 2009. The average number of observations per respondent was 5.9 during the study period. The average age of the respondents was 46.9 years; 89.1% of the study sample had no missing values in any measured indicator, and 94.5% had no missing values, except for the retrospective indicator for hospital days (see below).

**Table 1:** Descriptive statistics of the GSOEP sample

**Variable**	**Valid cases**	**Missing values**	**Sample**	**Weighted**
***Gender***		0.0%		
Male	131,408		47.9%	47.9%
Female	142,752		52.1%	52.1%
*Age*		0.0%		
18-29 years	51,134		18.7%	16.7%
30-44 years	80,552		29.4%	27.3%
45-64 years	91,685		33.4%	32.4%
65 years and over	50,788		18.5%	23.6%
*Timeframe*		0.0%		
1994-1998	63,691		23.2%	24.6%
1999-2003	90,993		33.2%	24.9%
2004-2006	61,294		22.4%	25.2%
2007-2009	58,182		21.2%	25.4%
*Income position*		0.1%		
< 60%	27,790		10.1%	12.5%
60-150%	186,273		68.0%	68.0%
150% and more	59,808		21.8%	19.5%
*Number of doctor visits per year*	261,115	4.8%	2.6	2.8
*Number of days in hospital per year*	249,477	9.0%	1.8	2.8
*Officially acknowledged disability (yes)*	273,107	0.4%	11.3%	13.6%
*General health status good/very good*	273,314	0.3%	50.0%	46.0%
*Satisfaction with health (1–10)*	273,159	0.4%	6.6	6.4

Data: GSOEP 1994–2010.

Valid Cases: Number of cases in category of categorical variable or with non-missing observations for count variables. Missing Values: Proportion of missing values by variable. Sample: mean or proportion in/of sample; weighted: mean or proportion in/of population as estimated by weighted sample * Data are collected retrospectively in the GSOEP, so a higher number of missing values is inevitable.

The GSOEP does not allow for the analysis of health care utilization by children and youth. As a consequence, we used additional data from the German Health Interview and Examination Survey for Children and Adolescents (KiGGS 2003–06), conducted between 2003 and 2006, to report utilization for male and female participants aged from 0 to 17 years [13]. The study data have been used to describe socioeconomic inequalities in health status as well as German health care utilization [14,15]. The data include indicators for doctor visits and hospital days, and income position based on their parent’s responses; these have been largely comparable with those reported in the GSOEP. Unfortunately, only the first wave of the study is currently available. Because of this, we have calculated the doctor consultations and hospital days by income position, age and gender using KiGGS 2003–06 and used them as approximation for the whole observational period. The sample consisted of 17,641 children (mean age 8.5 years; 50.9% male). The survey was approved by the Federal Office for Data Protection and by the Charité-Universitätsmedizin Berlin ethics committee.

The main variable of interest was the household income of the respondents after social transfers. We transformed the variable according to European and German standards for poverty reporting. The at-risk-of-poverty-rate is defined as the percentage of individuals living in households, where the total equalized household income is below 60% of national equalized median income, after social transfers [16]. We compared three groups including: the at-risk-of-poverty group (< 60% of the median), the middle income group (60% to 150%), and the relatively well-off (150% and above). In the KiGGS 2003–06 KiGGS study comparable income information was obtained from the participants’ parents.

We used the number of doctor visits and the number of days in a hospital (both measured annually) as indicators for in-patient and out-patient care. The indicator for number of doctor visits per year was based on self-reported doctor consultations during the 3 months prior to the survey, i.e. ‘Have you gone to a doctor within the last three months? If yes, please state how often’. The mean number of visits was 2.6 visits (99th percentile = 20); this value was multiplied by four to reflect the annual number of visits. The number of hospital days per year was asked retrospectively in successive (adjacent) panel waves. As a result, we were only able to cover years 1994 to 2009, although we used GSOEP data up to 2010. These data also had a higher number of missing values owing to the combined effects of item-non-response and drop out in subsequent waves. This indicator was based on two questions: ‘How many nights altogether did you spend in the hospital last year?’ (mean = 0.168 nights; 99th percentile = 2) together with ‘And how often were you admitted to a hospital in the year [last year]?’ (mean = 1.7 times; 99th percentile = 35). Both counts were summed to obtain the number of hospital days per year. In the KiGGS study, the same questions were used but were answered by the children’s parents.

The top-down projection of direct costs resulting from health inequalities was based on two alternative scenarios. The first scenario assumes that the whole population uses the same amount of resources as the highest income group. The second scenario assumes that it is at least possible to reduce the need of the lowest income group to the level of the middle income group. For the top-down projection we estimated the average costs per doctor consultation and per hospital day for the four periods. An average for both sectors was derived from the sum of expenditures divided by the average total number of doctor visits and hospital days. To estimate the totals we used the averages estimated for both indicators (by period, age and gender) and multiplied these by the total number of residents in the respective cells using official population statistics. This top-down approach is a useful instrument to simplify estimations of complex cost-generating processes. Further, we assumed that the direct costs in both sectors were equivalent to the total number of utilizations. The resulting approximations for unit costs are presented in Table 2.

**Table 2:** Top-down approximation of costs per doctor visit and per hospital day

	**Projected utilization based on GSOEP and KiGGS**		**Costs per sector according to Federal Statistical Office**		**Unit costs**	
	**Doctor visits mil. per year**	**Hospital days mil. per year**	**Ambulatory in mil. euros per year**	**Clinical in mil. euros per year**	**Ambulatory costs per visit**	**Clinical costs per day**
**Period**						
1994-1998	867.5	150.1	87,709	70,688	€ 101.10	€ 470.86
1999-2003	803.4	141.3	101,608	78,477	€ 126.48	€ 555.45
2004-2006	764.8	132.4	116,698	87,335	€ 152.59	€ 659.73
2007-2009	767.0	120.8	131,740	94,678	€ 171.75	€ 783.54

Data: GSOEP 1994–2010, KiGGS 2003–06, Federal Statistical Office 2011.

### Statistical analyses

All statistical analyses were based on a pooled dataset, including all panel waves from 1994 to 2010 to allow for trend analysis at the individual and population level. Analyses were performed using STATA 12.0 [17]. The study period was split into four consecutive periods (1994–1998, 1999–2003, 2004–2006, 2007–2009) that are roughly in line with governmental periods in Germany as well as the KiGGS study’s observational period (first wave); the number of doctor visits and the number of hospital days were then analyzed by income position, age and gender. Additionally, age-adjusted incidence rate ratios by income were computed using negative binomial regression for count outcomes with overdispersion. Confidence intervals were calculated based on robust Huber-White estimates to control for the data’s panel structure [18,19]. We then predicted the direct costs of inequalities in health care utilization based on the utilization of doctors and hospitals, and the total amount of costs in the ambulatory and hospital settings. Reference data for the analysis of direct costs were obtained from the Federal Statistical Office of Germany [20]. We used data of the so called ‘illness cost assessment’ on the total illness costs by sector and year.

## Results

**Figure 1:**
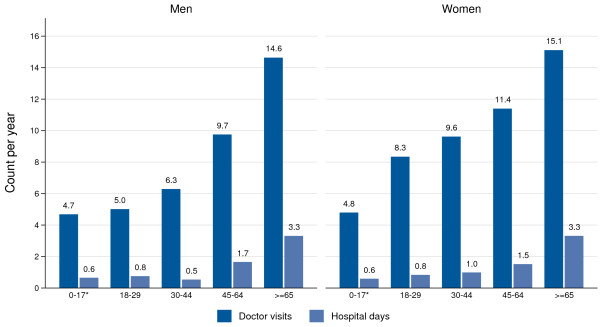
**Number of doctor visits and hospital days per year by age and gender in 2007 to 2009.** Data: GSOEP 2007–2010 for age 18+, * KiGGS-Study 2003–06 for age < 18.

Figure 1 shows the average number of doctor consultations and hospital days per year differentiated by age and gender for 2007 to 2009. Both indicators show a remarkable increase versus the age of the population. Overall, men and women aged 18 years or older visited a doctor an average of 9.2 and 11.3 times, respectively, and spent 1.6 and 1.8 days in hospital per year, respectively. On average, every additional year of age increases the number of doctor consultations per year by 1.5% for men and by 1.7% for women, and the number of hospital days per year by 3.5% for both sexes.

**Figure 2:**
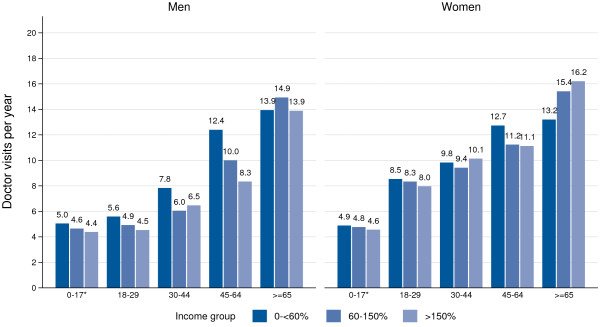
**Number of ambulatory doctor visits per year by age and income in 2007 to 2009.** Data: GSOEP 2007–2010 for age 18+, * KiGGS-Study 2003–06 for age < 18.

**Figure 3:**
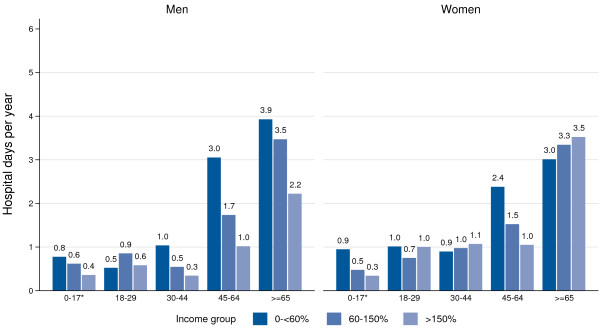
**Number of hospital days per year by age and income in 2007 to 2009.** Data: GSOEP 2007–2010 for age 18+, * KiGGS-Study 2003–06 for age < 18.

Figures 2 and 3 present the differences in doctor consultations and hospital days per year, for 2007 to 2009, differentiated by income position. During this timeframe, the differences relating to the number of doctor consultations were relatively low. Men in the lowest income group aged 18 years or older, reported 10.0 visits a year to a doctor, while those in the highest income group had 8.5 visits. The differences were especially marked for men aged 45 to 64 years, and also for male children and youths. By contrast, for women, the differences were small for doctor consultations by income. Having controlled for age, adult men and women in the lowest income group had a statistically significant 1.28-fold and 1.08-fold higher rate of ambulatory visits, respectively, when compared with those in the highest income group (Appendix); men and women also had a 2.22-fold and 1.33-fold higher rate of hospital days per year, respectively, than those in the highest income group (Appendix). The differences for children and youth were analyzed separately using the KiGGS-Study. The corresponding differences were shown to be 1.12-fold and 1.07-fold higher, and 1.85-fold and 2.86-fold higher, respectively (Appendix).

In Table 3, the results of the extrapolation from average health care utilization in the GSOEP and KiGGS to overall health care utilization and costs are presented. We compared the results of the extrapolation with the costs of two alternative scenarios: 1) all income groups share the age and gender specific health status and health care utilization rates of the highest income group; 2) the lowest income group can be leveled with the health status and utilization rates of the middle income group, while the utilization of the middle and high group remains as extrapolated. Overall, the results show that inequalities in health status and health care utilization in Germany are associated with considerable direct costs. The amount of the associated costs depends largely upon the underlying scenario that is used. The best case scenario (i.e. the whole population is as healthy and uses an equivalent amount of resources as the well off) would have hypothetically reduced the costs of health care by 19 to 25 billion euros per year (1.0% to 1.4% of German GDP in the respective years). The more realistic scenario (i.e. ‘closing the gap’ between the poor and middle income groups) would have led to a reduction of a mere 2 to 5 billion euros per year (0.1% to 0.2% of German GDP in the respective years). The higher costs for men than for women correspond with results for income inequalities in Germany regarding morbidity and mortality, that also tends to be greater for men than for women [21].

**Table 3:** Extrapolation of absolute numbers and simulation of costs for ambulatory and hospital care

	**Extrapolation of empirical values**		**Change in direct costs: Scenario I (all like 150%)**			**Change in direct costs: Scenario II (< 60% like 60-150%)**		
	**Doctor visits in mil. per year**	**Hospital days mil. per year**	**Ambulatory in mil. euros**	**Hospitals in mil. euros**	**Overall in mil. euros**	**Ambulatory in mil. euros**	**Hospitals in mil. euros**	**Overall in mil. euros**
**Men**								
1994-1998	355.8	65.3	-3,985	-10,893	**-14,878**	-322	-967	**-1,288**
1999-2003	336.6	64.9	-3,005	-9,112	**-12,117**	-132	-958	**-1,089**
2004-2006	326.5	62.7	-5,859	-15,131	**-20,990**	-824	-1,714	**-2,537**
2007-2009	332.3	55.7	-4,167	-15,460	**-19,627**	-755	-1,431	**-2,186**
**Women**								
1994-1998	511.7	84.8	-689	-2,936	**-3,625**	-618	-1,494	**-2,112**
1999-2003	466.7	76.4	-3,189	-3,300	**-6,489**	-554	-198	**-753**
2004-2006	438.3	69.7	1,313	-5,028	**-3,714**	-454	-2,324	**-2,778**
2007-2009	434.8	65.2	1,496	-2,140	**-644**	146	-1,231	**-1,085**
**Both**								
1994-1998	867.5	150.1	-4,674	-13,829	**-18,502**	-939	-2,461	**-3,400**
1999-2003	803.4	141.3	-6,194	-12,412	**-18,607**	-686	-1,156	**-1,842**
2004-2006	764.8	132.4	-4,546	-20,159	**-24,705**	-1,277	-4,038	**-5,315**
2007-2009	767.0	120.8	-2,671	-17,600	**-20,271**	-609	-2,662	**-3,271**

Data: SOEP 1994–2010, KiGGS 2003–06, Federal Statistical Office (2011a,b).

## Discussion

The aim of this study was to provide trends and cost scenarios for inequalities in health care utilization in Germany between 1994 and 2009. Overall, the results show that populations in Germany with low disposable incomes are using in-patient and out-patient health care more frequently than parts of the population who are better off. Every year, considerable costs are associated with the increased care needs of people who belong to an income group that is at risk of poverty. However, the results of the costs projections are based on the assumption that doctor visits and hospital days can be treated as indicators to estimate costs in the ambulatory and the hospital sector, which limits their generalizability.

The results presented here are in line with previous studies on inequalities in health care in Germany [22]. The main area of interest for previous studies was the number of doctor consultations comparing general practitioners and specialists [23-28]. These studies have shown that men and women with a lower socioeconomic status have more consultations than those with a higher status. Results of international comparative studies that have included Germany also have shown that the magnitude of inequities is near the average of other developed market economies [5,6]. Currently, there are only limited data on direct costs of health inequalities, notwithstanding the study of Mackenbach and colleagues [7]. In Germany, a study using health insurance company data showed that during the late 1990s, expenditure per insured person varied significantly depending on their personal income [29]. An increase in annual income of 5,000 euros—with age, sex, marital and disability status held constant—lowered the annual expenditure by approximately 175 euros.

In contrast to studies that are based on insurance data, this study used self-reported health care utilization and a top-down approximation of associated costs. The study has several limitations owing to the method of cost estimation and the dataset. To obtain sufficient file sizes for the analysis of time trends involving consultations, we opted to combine study data covering several years to maximize the statistical power for the cost estimation. The data from the GSOEP did not allow us to distinguish the number of visits to general practitioners and specialists; this would have been desirable because the methodology would have aligned with previous research approaches. A linkage between the survey data about socioeconomic position and process data on expenditures for each study member was not possible owing to the strict data protection laws in Germany. Additionally, we had to use data from the KiGGS-Study conducted in 2003 to 2006 for all periods compared. Therefore, our cost projections aren’t able to cover changes in inequalities in health care utilization among children and youth.

In order to assess the plausibility of the extrapolation and simulation regarding the costs of health inequalities, we compared the extrapolation results using absolute numbers of doctor consultations and hospital days with data from the Federal Statistical Office. It is known that self-reports about doctor consultations in Germany tend to underestimate the number of billed consultations. This is due to peculiarities of the medical system, where a physical visit to a doctor often leads to several billed contacts for that particular doctor in the books of the health insurance company [30]. Based on the GSOEP data for the time period 2007 to 2009, the average person in Germany had 10.4 doctor visits per year. In contrast, Barmer GEK, one of the largest German health insurance companies, had an average 18.7 contacts recorded in their files; this results in an underestimation of billed contacts by about 44% when using self-reports. An underestimation is also present for hospital days; the absolute numbers can be obtained by using hospital statistics of the Federal Statistical Office [31]. According to this Office, Germans spent a total of 142.4 million days in hospital in 2009, while the extrapolation of the GSOEP data computed 120.6 million days for 2007 to 2009, or approximately 15% less. In our opinion, this underestimation of doctor visits in the GSOEP should be regarded as negligible, because it is a systematic error that influences the absolute number of visits in all income groups in the same way, and hence not the relative differences between them. On the other hand, the underestimation of hospital days is likely to have influenced the results. Based on the analysis of panel mortality in the GSOEP, it is known that hospital days and a low socioeconomic status are positively associated with panel attrition [32]. Therefore, it can be argued that the observed differences between the income groups are conservative, in relation to the hospital days and resulting cost projections, because they underestimate the situation within the population.

## Conclusions

This study shows that inequalities in health have a major impact on the health care system in Germany and that they produce considerable costs for the sector. Despite an overall reduction in the number of age-specific doctor visits and hospital days over the last 15 years, inequalities were observed in all time periods that were analyzed. It is increasingly acknowledged that health inequality is avoidable and not a restricted trait of modern welfare states [2]. Therefore, the results of this study are a reminder for the need of an effective policy to close the health gap.

It would have been better to have had available more specific estimates of the direct costs of health inequalities in Germany, which could have been based on health insurance company data. Additional research is also needed to analyze the indirect costs of health inequalities in this country. In Germany, the key problem affecting this research area is the limited availability of data about socioeconomic differences in mortality. In relation to the results on inequities in out-patient care, their reasons and structural determinants should be investigated further in order to ensure that every patient receives the correct amount of treatment they need.

## Ethical approval

This study used anonymized secondary data of scientific use files, no ethical approval was needed.

## Appendix

In Tables 4 and 5 results negative binomial regression models for determinants of doctor visits and hospital days are shown. The models are controlled for age, adult men and women in the lowest income group had a statistically significant 1.28-fold and 1.08-fold higher rate of ambulatory visits, respectively, when compared with those in the highest income group; men and women also had a 2.22-fold and 1.33-fold higher rate of hospital days per year, respectively, than those in the highest income group (Table 4). The differences for children and youth were analyzed separately using the KiGGS-Study. The corresponding differences were shown to be 1.12-fold and 1.07-fold higher, and 1.85-fold and 2.86-fold higher, respectively (Table 5).

**Table 4:** Negative binomial regression model for doctor visits and hospital days (adults)

	**Men (n = 112,800)**				**Women (n = 123,365)**			
	**Doctor visits**		**Hospital days**		**Doctor visits**		**Hospital days**	
**Age**	OR	95%-CI	OR	95%-CI	OR	95%-CI	OR	95%-CI
18-29	Ref.		Ref.		Ref.		Ref.	
30-44	1.20*	[1.14,1.26]	1.21	[0.94,1.52]	1.10*	[1.06,1.14]	1.09	[0.97,1.23]
45-64	1.98*	[1.88,2.07]	2.97*	[2.38,3.70]	1.40*	[1.35,1.45]	1.59*	[1.42,1.77]
≥65	2.82*	[2.69,2.96]	5.79*	[4.62,7.25]	1.88*	[1.82,1.95]	3.52*	[3.17,3.89]
**Time period**								
1994-1998	Ref.		Ref.		Ref.		Ref.	
1999-2003	0.92*	[0.88,0.95]	0.96	[0.86,1.07]	0.89*	[0.87,0.92]	0.87*	[0.80,0.94]
2004-2006	0.86*	[0.82,0.89]	0.85*	[0.75,0.98]	0.82*	[0.79,0.84]	0.75*	[0.68,0.83]
2007-2009	0.86*	[0.83,0.90]	0.73*	[0.62,0.85]	0.81*	[0.79,0.84]	0.71*	[0.64,0.78]
**Income position**								
< 60%	1.28*	[1.20,1.37]	2.22*	[1.91,2.58]	1.08*	[1.03,1.12]	1.33*	[1.19,1.50]
60%- < 150%	1.14*	[1.11,1.18]	1.56*	[1.41,1.73]	1.01	[0.98,1.04]	1.13*	[1.03,1.23]
≥150%	Ref.		Ref.		Ref.		Ref.	
**Statistics**								
chi2	3,125		1,079		2,076		1,110	
p	0.000		0.000		0.000		0.000	

Data: SOEP 1994–2010.

*OR* Odds ratios [95% CI]: 95% confidence interval of the odds ratios based on robust standard errors. Ref.: reference category of indicator variables. All indicator variables are coded 0 = no and 1 = yes. p represents the p value for the likelihood-ratio test for the respective model, chi2 is the corresponding chi2 test, n the number of observations. * p < 0.05.

**Table 5:** Negative binomial regression model for doctor visits and hospital days (children)

	**Boys (n = 8,260)**				**Girls (n = 8,004)**			
	**Doctor visits**		**Hospital days**		**Doctor visits**		**Hospital days**	
	OR	95%-CI	OR	95%-CI	OR	95%-CI	OR	95%-CI
**Age**								
0-2	1.80*	[1.69.1.92]	2.47*	[1.55.3.94]	1.77*	[1.66.1.89]	2.70*	[1.64.4.45]
3-6	1.55*	[1.46.1.65]	0.90	[0.59.1.36]	1.41*	[1.33.1.50]	1.06	[0.67.1.66]
7-10	1.08*	[1.02.1.15]	1.07	[0.70.1.63]	1.02	[0.97.1.09]	1.28	[0.82.2.00]
11-13	Ref.		Ref.		Ref.		Ref.	
14-17	1.10*	[1.04.1.17]	1.27	[0.83.1.92]	1.43*	[1.35.1.51]	2.94*	[1.90.4.54]
**Income position**								
< 60%	1.12*	[1.05.1.21]	1.85*	[1.12.3.06]	1.07*	[1.00.1.15]	2.86*	[1.68.4.88]
60%- < 150%	1.05	[0.99.1.12]	1.48	[0.93.2.36]	1.04	[0.98.1.11]	1.18	[0.72.1.92]
≥150%	Ref.		Ref.		Ref.		Ref.	
**Statistics**								
chi2	566	35	486	66				
p	0.000	0.000	0.000	0.000				

Data: KiGGS 2003-06.

*OR* Odds ratios [95% CI]: 95% confidence interval of the odds ratios based on robust standard errors. Ref.: reference category of indicator variables. All indicator variables are coded 0 = no and 1 = yes. Under regression diagnostics, p represents the p value for the likelihood-ratio test for the respective model. * p< 0.05.

## Competing interests

The authors declare that they have no competing interest.

## Authors’ contributions

LEK planned and wrote the first version of the manuscript and did all the statistical analysis. TL assisted in planning the manuscript and the analysis and revised the first draft of the manuscript. Both authors read and approved the final manuscript.

## Pre-publication history

The pre-publication history for this paper can be accessed here:

http://www.biomedcentral.com/1472-6963/13/271/prepub
